# Depression Increases Sympathetic Activity and Exacerbates Myocardial Remodeling after Myocardial Infarction: Evidence from an Animal Experiment

**DOI:** 10.1371/journal.pone.0101734

**Published:** 2014-07-18

**Authors:** Shaobo Shi, Jinjun Liang, Tao Liu, Xiaoran Yuan, Bing Ruan, Lifang Sun, Yanhong Tang, Bo Yang, Dan Hu, Congxin Huang

**Affiliations:** 1 Department of Cardiology, Renmin Hospital of Wuhan University, Wuhan, China; 2 Cardiovascular Research Institute, Wuhan University, Wuhan, China; 3 Masonic Medical Research Laboratory, Utica, New York, United States of America; University Hospital of Würzburg, Germany

## Abstract

Depression is an independent risk factor for cardiovascular events and mortality in patients with myocardial infarction (MI). Excessive sympathetic activation and serious myocardial remodeling may contribute to this association. The aim of this study was to discuss the effect of depression on sympathetic activity and myocardial remodeling after MI. Wild-type (WT) rats were divided into a sham group (Sham), a myocardial infarction group (MI), a depression group (D), and a myocardial infarction plus depression group (MI+D). Compared with controls, the MI+D animals displayed depression-like behaviors and attenuated body weight gain. The evaluation of sympathetic activity showed an increased level in plasma concentrations of epinephrine and norepinephrine and higher expression of myocardial tyrosine hydroxylase in the MI+D group than the control groups (p<0.05 for all). Cardiac function and morphologic analyses revealed a decreased fractional shortening accompanied by increased left ventricular dimensions, thinning myocardium wall, and reduced collagen repair in the MI+D group compared with the MI group (p<0.05 for all). Frequent premature ventricular contractions, prolonged QT duration and ventricular repolarization duration, shorted effective refractory period, and increased susceptibility to ventricular arrhythmia were displayed in MI+D rats. These results indicate that sympathetic hyperactivation and exacerbated myocardial remodeling may be a plausible mechanism linking depression to an adverse prognosis after MI.

## Introduction

The close, bidirectional relationship between depression and myocardial infarction (MI) is well established. Approximately 20% patients with MI have a major depressive disorder or depressive states [1], self-reported symptoms of depression are associated with 1.31 times risk of incident acute MI [2], and a history of MI is an independent risk factor of in-hospital depressive symptoms [3]. Post-infarction depression has been associated with a worse prognosis. A recent meta-analysis showed that depression following MI was independently associated with adverse prognosis, with a 22% increased risk of all-cause mortality and a 13% increased risk of cardiovascular events after adjustment for cardiac disease severity [4]. A previous meta-analysis suggested that post-MI depression is associated with a 1.6- to 2.7-fold increased risk of impaired cardiovascular outcomes within 24 months [5]. Depression is associated with abnormal cardiac electrophysiology features, such as prolonged QT duration and low heart rate variability [6], and significantly increases the risk of ventricular arrhythmia (VA) and sudden cardiac death (SCD) [7], [8].

However, the mechanism by which depression has adverse effects on the prognosis of MI patients remains unclear, which may be related to the sympathetic hyperactivity and exacerbation of cardiac structural and electoral remodeling. Therefore, the primary goal of this study is to test this hypothesis and to better understand the association between depression and MI.

## Materials and Methods

### Animals

All experiments were conducted in accordance with the Guide for the Care and Use of Laboratory Animals published by the U.S. National Institute of Health (NIH Publication No. 85–23, revised 1996). All animal studies were reviewed and approved by the animal experimental administration of Wuhan University, China. A total of 50 male Sprague Dawley rats (280–300 g) were randomly placed into a sham group (Sham group, n = 10), a myocardial infarction group (MI group, n = 15), a depression group (D group, n = 10), and a myocardial infarction plus depression group (MI+D group, n = 15). MI was created by ligation of the left anterior descending coronary artery, and the model of depression was induced with chronic mild stress (CMS) procedure. Sodium pentobarbital was used as an anesthetic, and the adequacy of anesthesia was confirmed by the absence of a withdrawal response to hind paw nociceptive stimulation.

### Ligation of the left coronary artery

Coronary artery ligation was performed as described previously [9]. In brief, rats were anesthetized (pentobarbital sodium, 40 mg/kg, i.p. Sigma), intubated and ventilated by a volume-constant rodent ventilator, and a left thoracotomy was performed. The heart was exteriorized from the thorax, and the left anterior descending artery was ligated using an 8-0 prolene suture between the right ventricular outflow tract and left atrium. The heart was then returned to its normal position and the thorax closed. The Sham group and D group were operated with the same protocol, except the coronary artery was not ligated.

### CMS procedure

According to previous methods [10], the D and MI+D groups were subjected to the following stressors: food deprivation immediately followed by 1 h of access to restricted food; water deprivation immediately followed by 1 h exposure to an empty bottle; continuous overnight illumination; cage tilt (45°) for 24 h; white noise for 12 h; restraint (a rat in a 10 cm×13 cm×13 cm plastic box with air holes) for 1 h; strobe light illumination for 12 h (120 flashes/min, 1 h); light cycle (continuous illumination) for 24 h; dark cycle (continuous darkness) for 24 h; damp bedding (200 ml water poured into the sawdust bedding) for 12 h; cold swim (0°) for 5 min. The depression model was designed to maximize the unpredictable nature of the stressors per day for 4 weeks.

### Sucrose preference test (SPT)

A SPT was used to determine anhedonia, one of the core symptoms of major depression in humans [11]. A two-bottle preference test was used, where all animals had access to both water and a 1% (w/v) sucrose solution for 1 h following 24 h food and water deprivation. Intake was measured by weighing pre-weighed bottles at the end of the test. To habituate animals to the sucrose solution, three habituation tests were performed, where animals had only access to sucrose solution for 24 h. Sucrose preference (SP) was calculated according to the following formula: SP  =  sucrose intake/(sucrose intake + water intake) ×100. The SPT was performed after 4 weeks of CMS.

### Open field test (OFT)

The spontaneous locomotor activity and behavioral response to a new environment was detected in an open field arena (100×100×40 cm^3^) during a 5-minute period [12], [13]. A video-tracking system (Ethovision 3.0, Noldus) was used to measure the locomotor activity of the animals, included horizontal movements (the travelled distance) and vertical movements (rearing), as well as the time spent in the center and periphery of the test arena. The OPT was also performed after 4 weeks of CMS.

### Evaluation of cardiac function

Cardiac function was evaluated by echocardiography under anesthesia (pentobarbital sodium, 40 mg/kg, i.p. Sigma), with spontaneous respiration. Two-dimensional short- and long-axis images of the left ventricle were obtained at the papillary muscle level (Vevo 770, Visual Sonics, Toronto, Ontario) [14]. The following parameters were measured: left ventricular end-diastolic dimension (LVEDD), left ventricular end-systolic dimension (LVESD), left ventricular wall thickness (LVWT), left ventricular ejection fraction (LVEF), and fractional shortening (FS). Three consecutive cardiac cycles were analyzed, and the average measurement was used for data analysis.

### Surface ECG recording

The surface electrocardiogram (ECG, lead II) was then recorded for each animal for 10 min (AD Instrument, PowerLab system) under anesthetic (pentobarbital sodium, 40 mg/kg, i.p. Sigma). The basic parameters were measured and analyzed: heart rate (HR), duration of QT and QTc intervals. The QT interval was corrected for heart rate with the Bazett's formula: QTc = QT/(RR/100)^1/2^.

### Evaluation of blood pressure and sympathetic activity

The mean blood pressure (BP) was measured by right carotid artery cannulation with a stretch polyethylene tube, and data were recorded using the Powerlab system and Chart 7.0 software (AD Instruments Ltd, Chalgrouve, UK). After the completion of BP recording, 1 ml of artery blood was collected to measure epinephrine (E) and norepinephrine (NE) excretion with high-performance liquid chromatography [15].

### Isolated heart preparation

The rats were anesthetized and heparinized for 10 min, tissues were harvested immediately, then perfused according to the Langerdorff technique [16] at constant pressure (70–90 cm water) with HEPES-buffered Tyrode's solution of the following composition (in mmol/L): NaCl, 135; KCl, 5.4; CaCl_2_, 1.8; MgCl_2_, 1; Na_2_HPO_4_, 0.3; HEPES, 10; and glucose, 10. The solution was adjusted to pH 7.4 with NaOH and ventilated with 95% oxygen and 5% carbon dioxide.Isolated hearts were perfused for 20 min before further experiment. Hearts that did not recover a regular spontaneous rhythm or that had irreversible myocardial ischemia would be discarded.

### Programmed electrical stimulation

Epicardial programmed electrical stimulation (PES) was performed as previously described [16]. In brief, two custom-made Ag-AgCl electrodes (diameter: 0.25 mm, spacing: 0.5 mm) were used to record monophasic action potentials (MAP) and paired platinum-stimulating electrodes (diameter: 0.25 mm, spacing: 1 mm) were positioned on the basal surface of the right ventricle. A programmed stimulation protocol was delivered with square-wave pulses of 2 ms and amplitudes of three times the diastolic threshold, consisting of 8 S_1_ pulses at four basal cycle lengths (CL: 300 ms, 250 ms, 200 ms and 150 ms) followed by a premature S_2_ pulse with progressively shorter S_1_-S_2_ interval steps: 300 to 100 ms in 20 ms steps, 100 ms to loss of capture in 10 ms steps, then last conducted S_2_ to effective refractory period (ERP) in 1 ms steps. All measured signals were recorded with a PowerLab system (AD Instruments Ltd, Chalgrouve, UK), and were amplified and filtered between 0.3 Hz and 1 kHz. The MAP waveforms were analyzed with Chart 7.0 software (AD Instruments Ltd, Chalgrouve, UK). The APD_90_ and ERP were measured at both of the left and right anterior basic ventricle. A VA was defined as more than three premature ventricular contractions (PVCs) and Sustained VA was defined as lasting ≥30 s. The VA threshold was evaluated with the CL of S_1_-S_2_ interval which first induced a VA.

### Infarct size determination

After the completion of electrophysiology experiments, the hearts were weighed and fixed in 4% formaldehyde for histologic analyses. Infarct size was determined as the mean percent of epicardial and endocardial circumference occupied by scar tissue observed upon Masson's trichrome staining (Sigma). Morphometric evaluation included quantification of LV epicardial and endocardial circumference, scar tissue circumference, infarct wall thickness and noninfarcted remote wall thickness. And the collagen composition of the scar and border zone was also determined. Four sections per heart, from 4 hearts in MI group and MI+D group, were analyzed and averaged.

### Immunohistochemistry

To investigate the spatial distribution and quantification of sympathetic nerve fibers, the immunohistochemical staining with tyrosine hydroxylase (TH: a marker for sympathetic nerve tissue) was performed on LV muscles from the border regions in MI group and MI+D group, and the relative regions from Sham group and D group were selected as controls. As described by previous methods [17], Paraffin-embedded sections were incubated with anti-TH (1∶200; Sigma) in 0.5% BSA in PBS overnight at 37°C. As a second antibody, anti-mouse lgG was conjugated to fluorescein isothiocyanate for TH. Slides were analyzed by two blinded reviewers using Image Pro Plus software. The fluorescent TH nerve fiber area was expressed relative to the total sectional cardiomyocyte area (% TH-immunoreactive area). Ten randomly selected fields per region from 4 hearts in every group were quantified and averaged.

### Statistical analysis

Statistical analysis was performed with SPSS software (version 17.0, SPSS, Inc. Chicago, Illinois). Continuous variables are expressed as the mean±SD. Proportions were reported as percentages. Test groups were compared using Student's *t*-test, chi-square test or ANONA test when appropriate, and the Least-significant difference (LSD) test or Tamhane's T2 test was performed as a post-hoc test following ANOVA. The survival rate was analyzed with Kaplan-Meier methods. A p value≤0.05 was considered to be statistically significant.

## Results

### Depressive-like behavior

**Figure 1:**
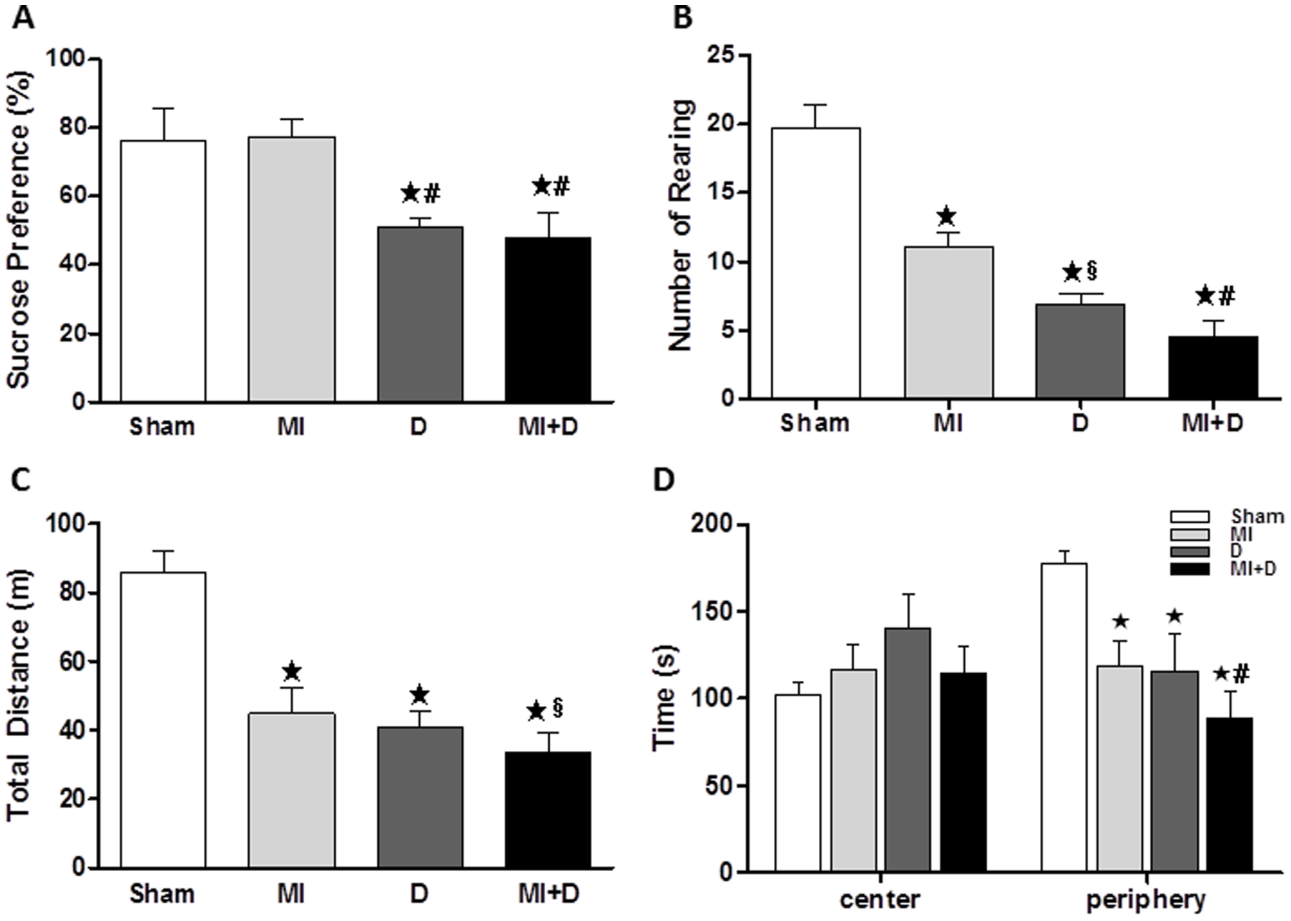
The depressive-like behaviors evaluated by the sucrose preference test and the open field test. After 4 weeks of chronic mild stress, the rats showed a decrease in sucrose preference (A) and deficiency in spontaneous locomotor activity, included rearing (B), travelled distance (C) and exploration activity (D). **^★^**p<0.01 vs Sham group, **^★^**p<0.01 and **^§^**p<0.05 vs MI group.

After 4 weeks of CMS, the SPT and the OFT were performed to assess depressive-like behaviors. The MI+D and D rats showed a significant decrease in SP compared with the controls (Sham, 76.12±9.43%, MI 77.26±5.17%, D 50.91±2.51%, MI+D 48.02±7.46%, p<0.01, Fig 1A). The SP was no different between the MI group and Sham group, which is considered as a symptom resembling anhedonia. The amount of rearing activity was less in the MI+D and D groups than the control groups (Sham, 19.70±1.74, MI 11.1±1.03, D 6.88±0.82, MI+D 4.55±1.07, p<0.01, Fig 1B). Additionally, the MI+D and D rats showed a decrease in the total travelled distance and the amount of time spent in the arena-periphery (Fig 1C and D), which are considered other primary depressive-like symptoms (i.e. reduced locomotor activity and exploratory activity).

### The survival rates and body weight

**Figure 2:**
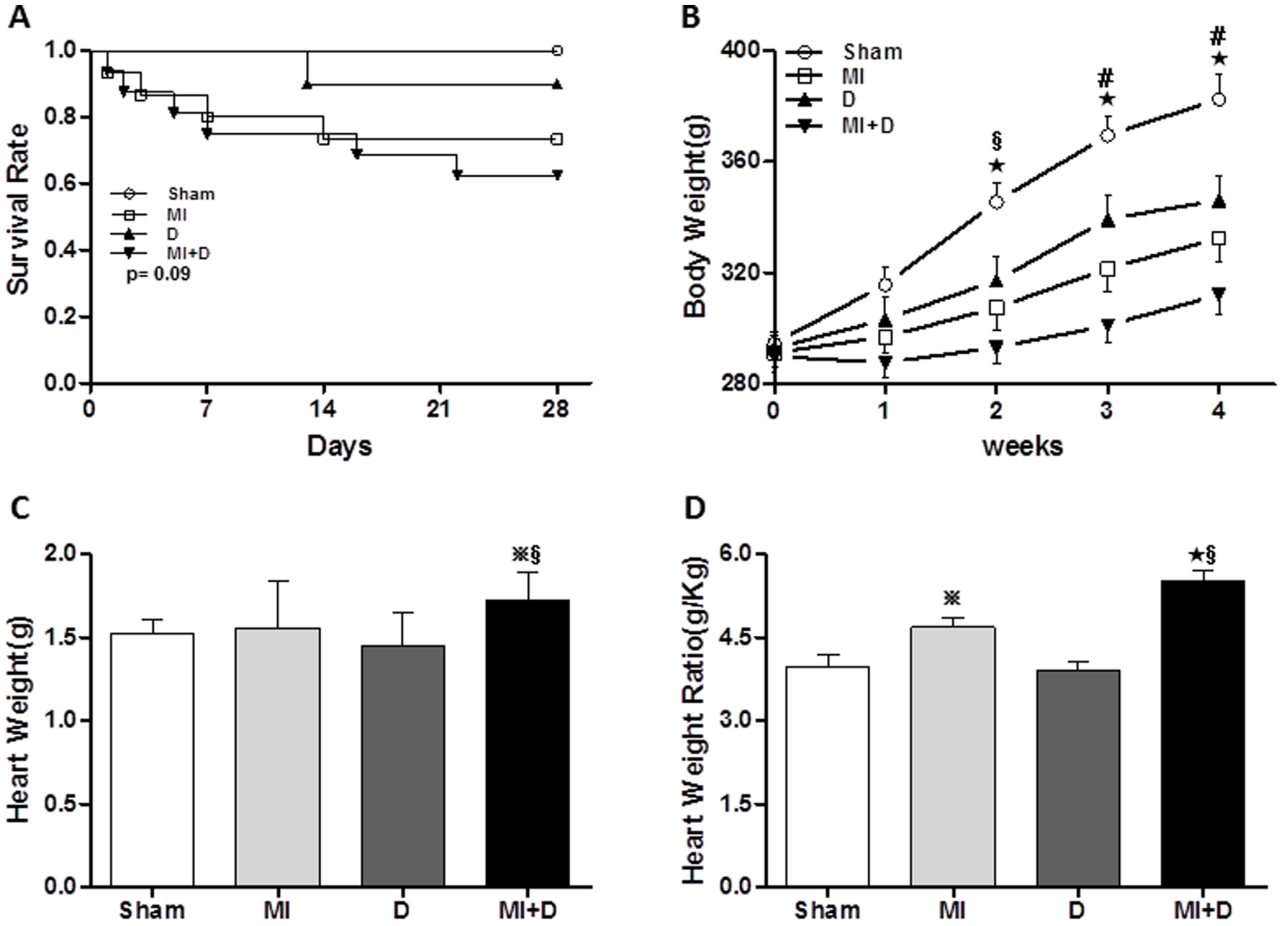
The comparisons in survival rate and body weight gain. (A), The survival rate was not different among groups (p = 0.09). The MI+D rats displayed an attenuated body weight gain (B), increased heart weight (C) and increased heart weight ratio (D). ^★^p<0.01 and **^
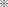
^**p<0.05 indicate MI+D group or MI group vs Sham group, **^#^**p<0.01 and **^§^**p<0.05 indicate MI+D group vs MI group.

The survival rates after MI and depression model preparation were as follows: Sham group, 100% (10/10); MI group, 73.3% (11/15); D group, 90% (9/10); MI+D group 60.0% (9/15). There was no significant difference for the survival analysis (p = 0.09, Fig 2A). The MI+D rats showed an attenuated body weight gain and overall body weight throughout the study (Fig 2B). Both absolute heart weight and relative heart weight (heart weight/body weight ratio) were greater in the MI+D group than the control groups (absolute heart weight: Sham 1.52±0.09 g, MI 1.56±0.28 g, D 1.45±0.20 g, MI+D 1.72±0.18 g, p<0.01, and relative heart weight: Sham 3.98±0.21 g/kg, MI 4.68±0.17 g/kg, D 3.89±0.16 g/kg, MI+D 5.50±0.20 g/kg, p<0.01, Fig 2C and D).

### Cardiac function

At 5 weeks after coronary artery ligation, the MI+D rats and MI rats exhibited increases in LVEDD, LVSDD and LVWT and decreases in FS compared with the controls. In addition, adverse remodeling (as manifested by increased LVEDD, LVSDD) was significant in the MI+D group compared with the MI group, and LV dysfunction (as manifested by decreases in FS) was more remarkable in the MI+D group than the MI group. There were no significant differences between the Sham and D groups. A summary of the data is shown in Table 1.

**Table 1:** Echocardiogram parameters of left ventricular structure and function.

	n	LVEDD(mm)	LVESD(mm)	LVWT(mm)	LVEF (%)	FS (%)
Sham group	10	4.74±0.26	2.45±0.28	1.23±0.76	78.01±2.13	26.54±2.02
MI group	11	6.21±0.78^★^	4.11±0.14^★^	1.64±0.42^★^	36.55±3.64^★^	18.32±1.29^★^
D group	9	4.82±0.46	2.75±0.82	1.27±0.43	76.31±3.54	25.84±2.14
MI+D group	9	6.82±0.21^★**§**^	4.51±0.23^★**★**^	1.91±0.83^★**§**^	34.22±2.36^★^	15.29±1.63^★**§**^

LVEDD, left ventricular end-diastolic diameter; LVESD, left ventricular end-systolic diameter; LVWT, left ventricular wall thickness; LVEF, left ventricular ejection fraction; FS, fractional shortening; ^★^p<0.01 vs Sham group, **^#^**p<0.01 and **^§^**p<0.05vs MI group.

### Changes in the surface ECG

**Figure 3:**
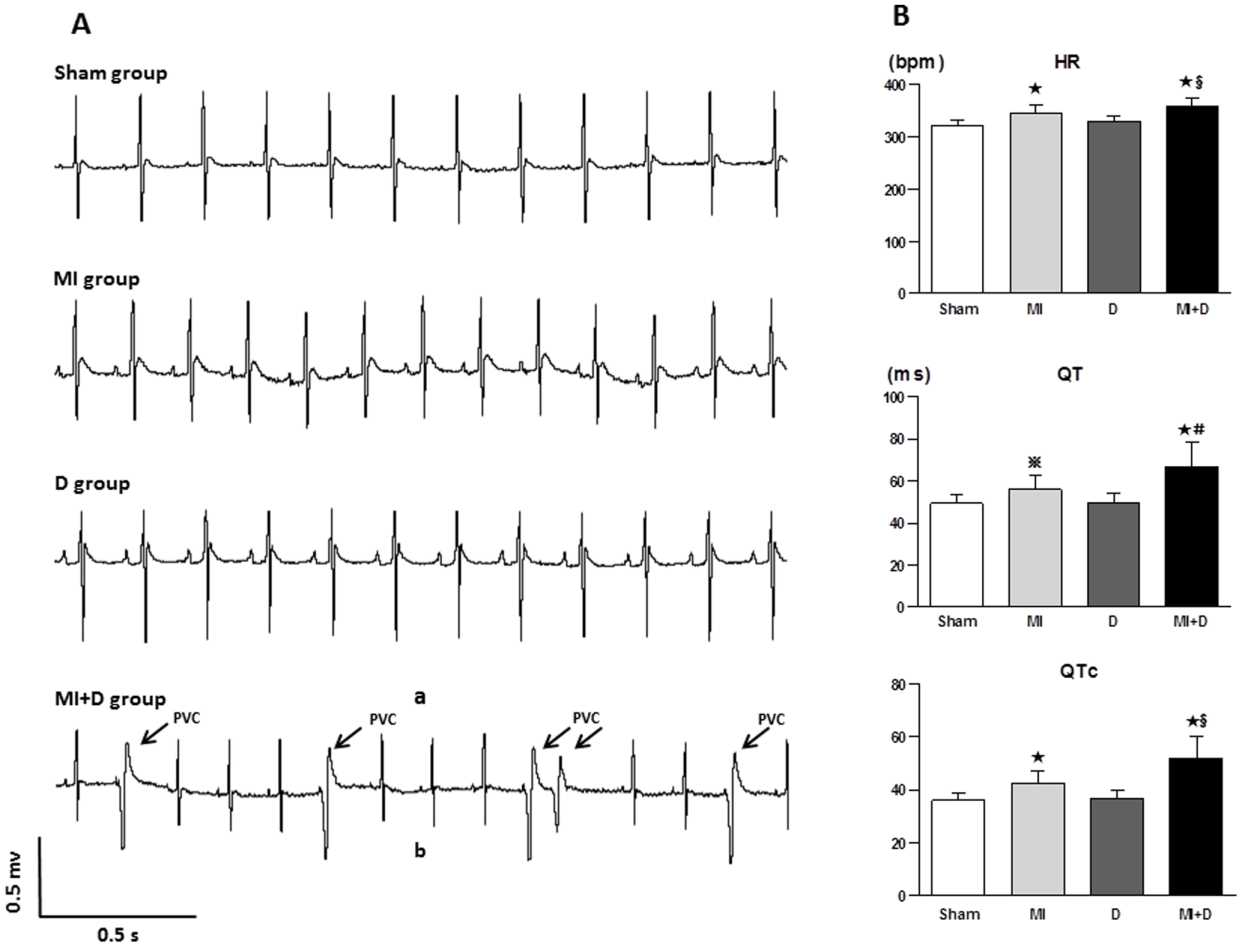
The representative ECG and the comparison in ECG parameters. (A), Frequent premature ventricular contraction were observed in a MI+D rat; none were founded in the control groups. (B), The faster HR, longer QT and QTc intervals were shown in the MI+D group than the MI group. ^★^p<0.01 and **^
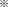
^**p<0.05 vs Sham group, **^#^**p<0.01 and **^§^**p<0.05 vs MI group.

Frequent PVCs were found in two MI+D animals under anesthesia, but none were found in the other groups (Fig 3A). Other ECG abnormalities, including elevated HR, prolonged QT and QTc interval, were observed in the MI+D and MI groups; however, these changes were significantly greater in the MI+D group than the MI group (Fig 3B).

### BP and sympathetic activity

**Figure 4:**
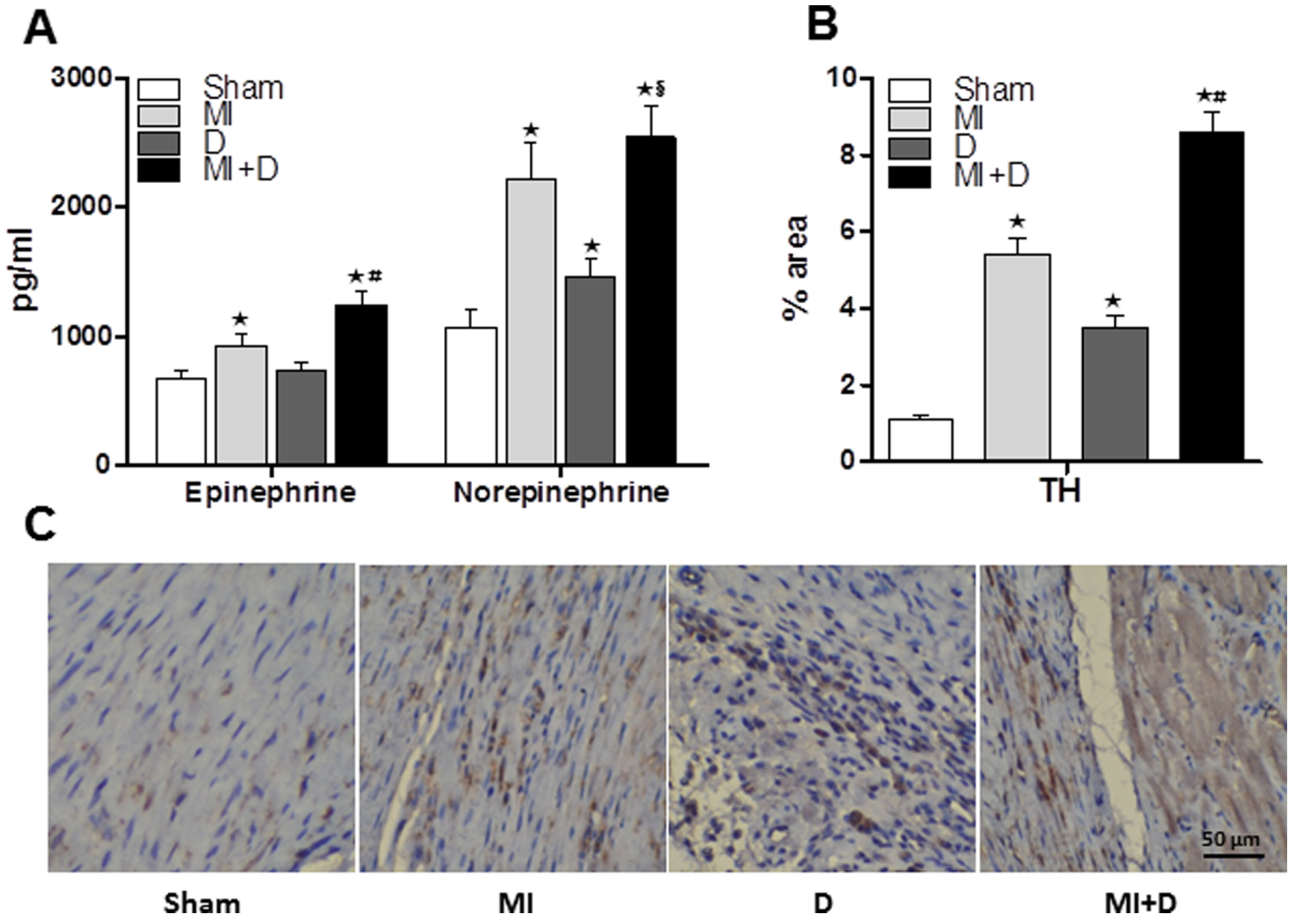
Quantification of plasma epinephrine and norepinephrine, cardiac tyrosine hydroxylase, and immunohistochemical staining for tyrosine hydroxylase nerve fiber. (A), The concentrations of epinephrine and norepinephrine were higher in MI+D rats than MI rats, but only norepinephrine was higher in D rats versus Sham rats. (B) and (C), Compared with MI rats, the sympathetic innervation of the border regions was particularly prominent in MI+D rats, and sympathetic hyperinnervation was also observed in D rats.^★^p<0.01 vs Sham group, ^#^p<0.01 and ^§^p<0.05 vs MI group.

BP was not significantly different among groups. However, the plasma level of E and NE were higher in the MI+D group than the MI group (E: 1243±102 pg/ml vs 932±84 pg/ml, p<0.01; NE: 2547±243 pg/ml vs 2215±283 pg/ml, p<0.05, Fig4A), which indicated that sympathetic activity was hyperactive after CMS. The MI+D rats and D rats expressed a higher percentage of TH-immunoreactive nerve fibers in ischemia border regions than the control rats (Sham: 1.1±0.13%, MI: 5.4±0.42%, D: 3.5±0.32%, MI+D: 8.6±0.54%, p<0.01, Fig4 B and C), indicating that cardiac sympathetic hyperinnervation was induced by 4 weeks of CMS.

### MAP analysis

**Figure 5:**
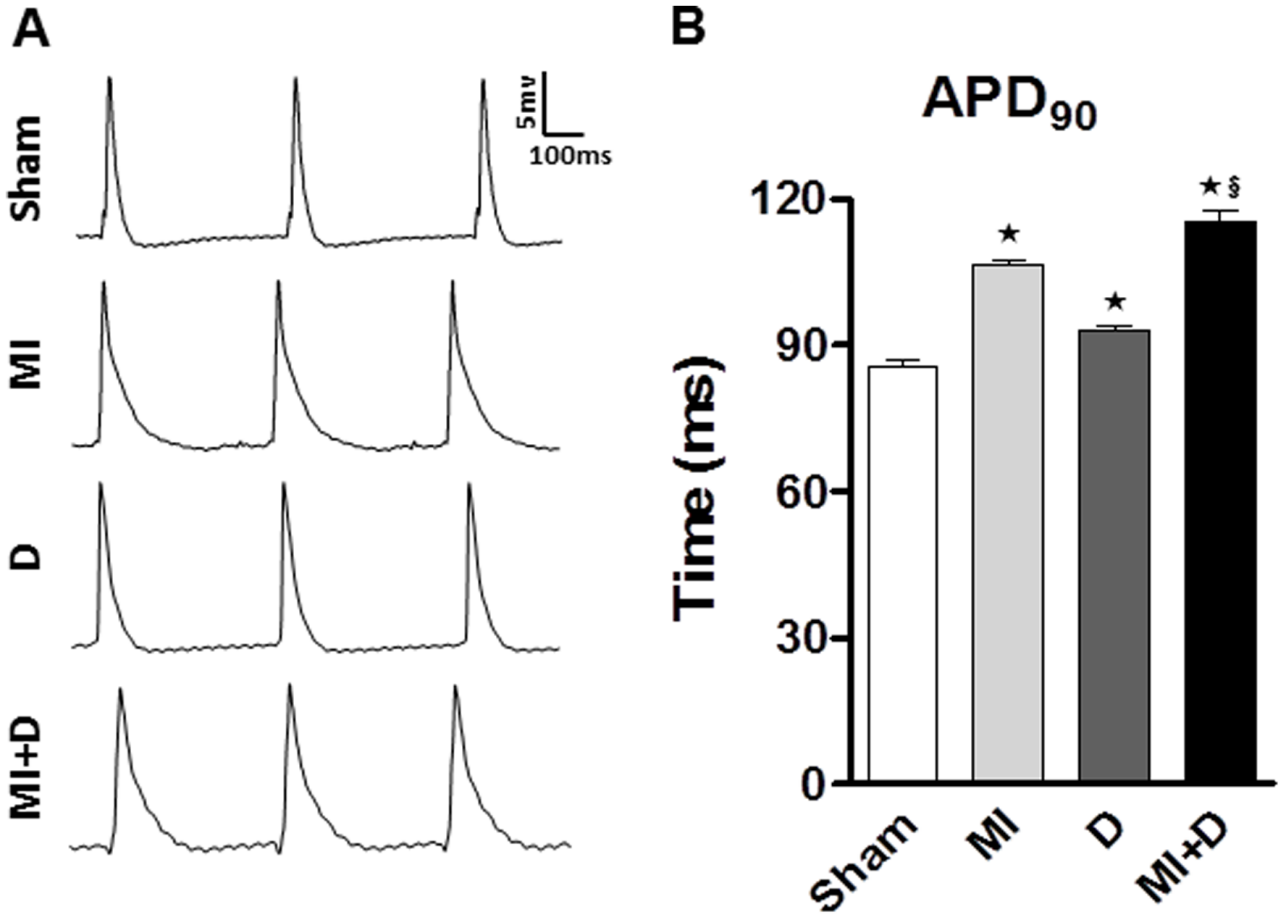
Spontaneous action potential durations (APD). (A), Representative monophasic action potentials recorded from LV. (B), Compared with controls, the APD_90_ was prolonged in MI+D rats and MI rats. ^★^p<0.01 vs Sham group, **^§^**p<0.05 vs MI group.

**Figure 6:**
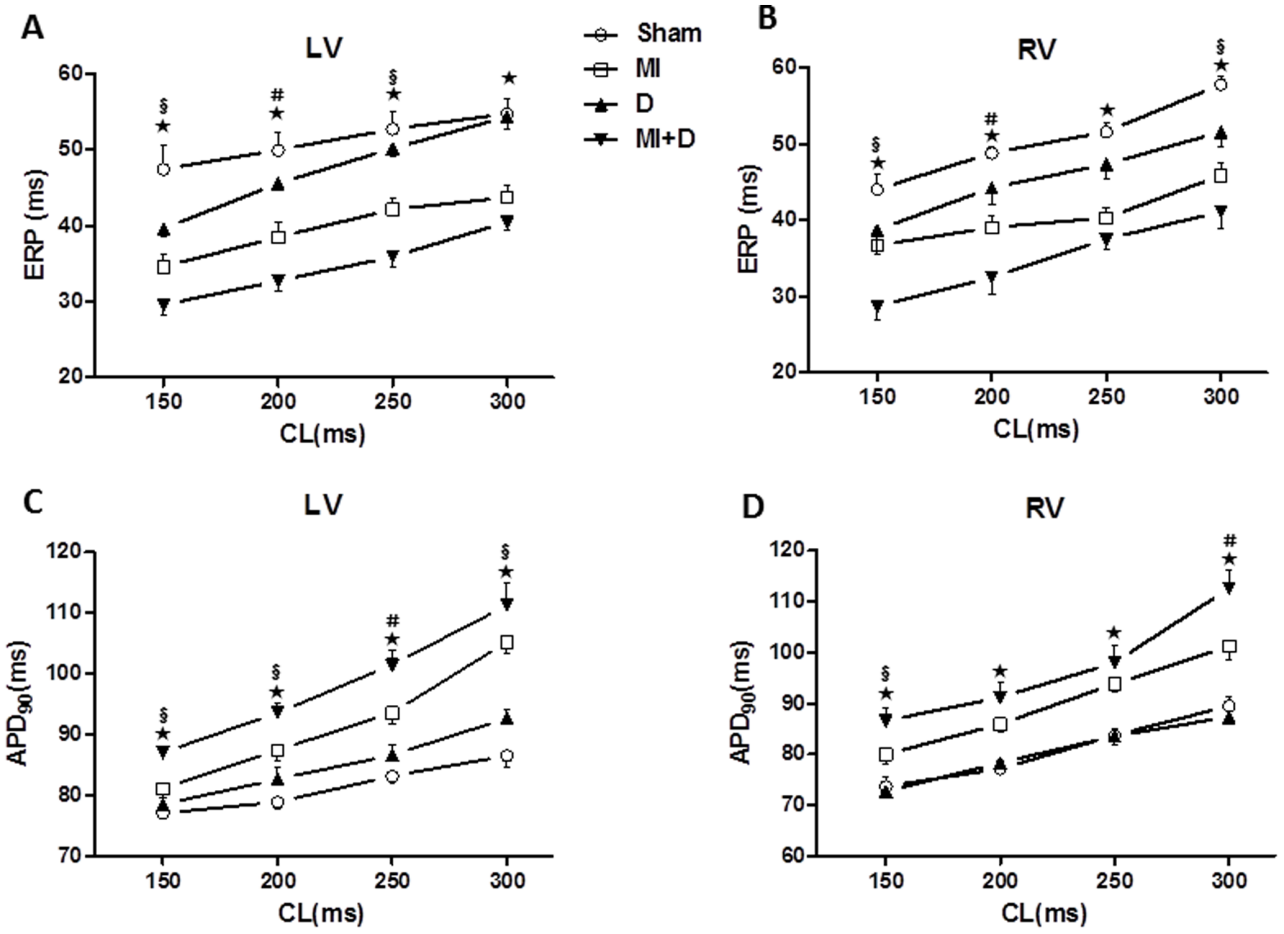
Restitution kinetics of effective refractory period (ERP) and action potential duration (APD). (A) and (B), Restitution kinetics measured from left ventricle (LV) and right ventricle (RV) for ERP. (C) and (D), Restitution kinetics measured from LV and RV forAPD_90_.^★^p<0.01 indicate MI+D group vs Sham group;**^#^**p<0.01 and **^§^**p<0.05 indicate MI+D group vs MI group.

As shown in Fig 5, the APDs recorded from spontaneous heart contractions were significantly prolonged in the MI+D group and MI group compared with the controls. While pacing at different CL, the APDs of LV and RV were all longer in the MI+D group than the controls, and the mean ventricular APDs were also significantly prolonged in the MI+D group compared with the MI group. In contrast to the trend of APDs, the ERPs were all shorter in the MI+D group than the MI group (Fig 6 and Table 2).

**Table 2:** The mean ventricular ERP and APD_90_ under various cycle lengths.

CL(ms)	ERP(ms)				APD_90_(ms)			
	Sham(n = 10)	MI(n = 11) ^★^	D(n = 9) ^★^	MI+D(n = 9) ^★§^	Sham(n = 10)	MI(n = 11) ^★^	D(n = 9) ^★^	MI+D(n = 9) ^★§^
300	56.3±2.1	44.5±1.5	52.9±1.1	39.8±1.4	81.9±1.4	103.2±2.1	90.0±1.4	111.9±2.6
250	52.2±2.6	41.0±1.4	48.7±0.8	37.2±1.3	78.9±1.0	93.7±1.7	85.1±1.2	99.7±1.8
200	49.9±2.5	38.4±2.0	44.9±1.1	32.6±1.4	74.9±1.2	85.7±1.5	78.3±1.0	92.3±2.3
150	45.7±2.3	35.4±1.6	39.1±1.5	29.1±1.0	73.5±1.3	80.5±1.1	71.7±1.1	86.8±2.4

CL, cycle length; ERP, effective refractory period; APD_90_, action potential duration at 90% recovery to baseline. ^★^p<0.01 vs Sham group; **^§^**p<0.05 vs MI group.

### VA vulnerability

**Figure 7:**
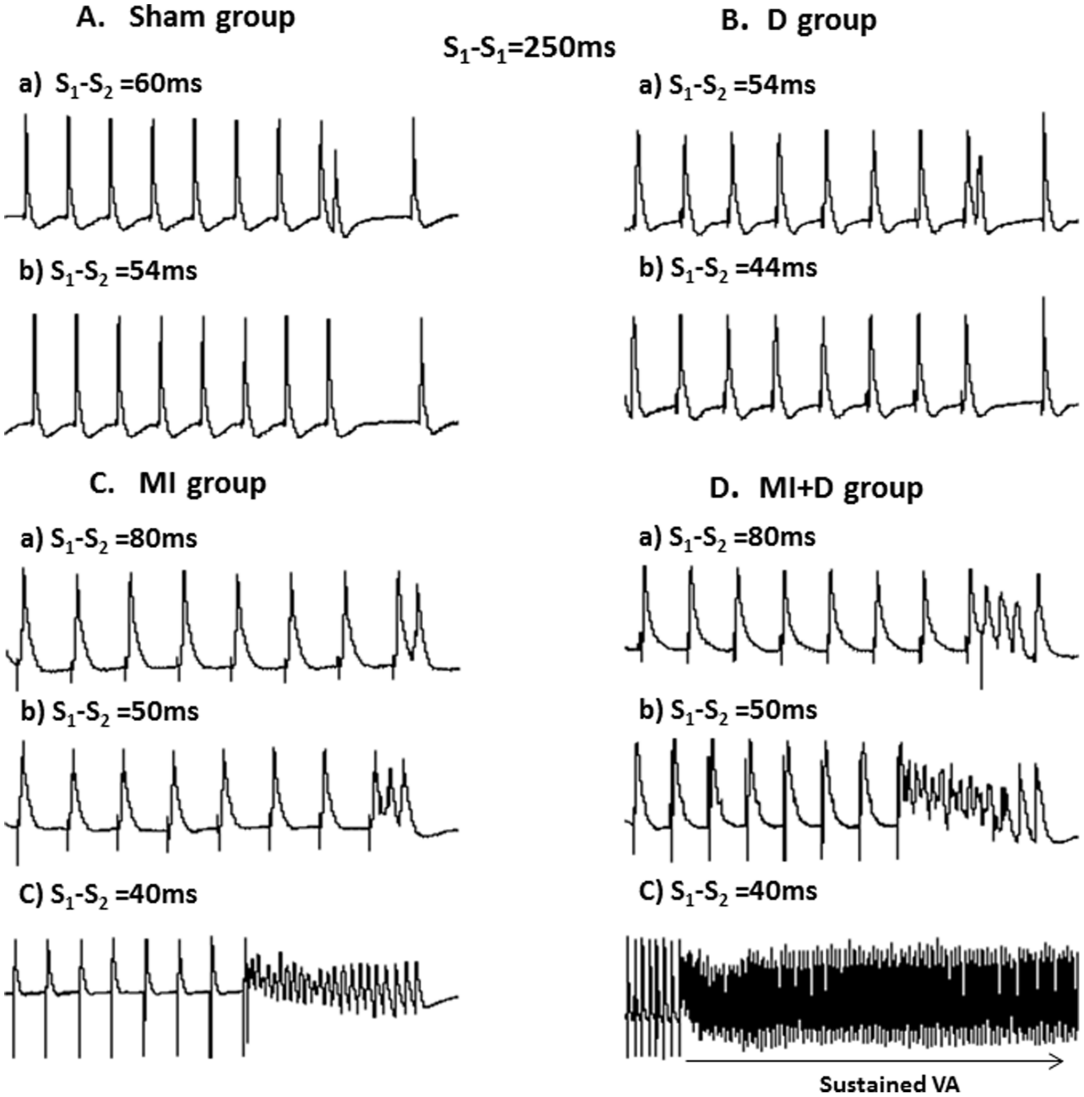
Inducibility of ventricular arrhythmia (VA) by programmed electrical stimulation. (A) and (B), Representative action potential (AP) traces from left ventricle (LV) in a Sham rat and a depression rat. No VA could be elicited by programmed stimulation. (C), Representative AP traces from LV in an MI rat. While pacing at S_1_-S_2_ of 80 ms, a normal AP was captured and propagated (a). An impulse was delivered at S_1_-S_2_ of 50 ms, and PVC was elicited (b). When delivered at shorter interval of 40 ms, a transient VA was induced (c). (D), Representative AP traces from an MI+D rat, while pacing at S_1_-S_2_ of 80 ms and 50 ms, transient VA was induced (a and b). When delivered at S_1_-S_2_ of 40 ms, a sustained VA was elicited (c).

VA was inducible in all of the MI and MI+D rats but none of the Sham and D rats (p<0.01). In the Sham and D rats, a premature impulse close to the ERP was captured and propagated, whereas shorter intervals failed to be captured and did not induce VA (Fig 7A and 7B). In the MI rats, a premature impulse at S_1_-S_2_ of 80 ms was captured and propagated normally, but a 50-ms S_1_-S_2_ interval induced PVC and a 40-ms S_1_-S_2_ interval induced transient arrhythmias (Fig 7C). However, in the MI+D hearts, an 80-ms S_1_-S_2_ interval started to induce a transient arrhythmias, and a shorter interval of 40 ms produced sustained VA (Fig 7D). VA was initiated at S_1_-S_2_ of 75±10 ms in the MI+D group, which was greater than the MI group (45± 15 ms, p<0.01).

### Infarct size and left ventricle morphometry

**Figure 8:**
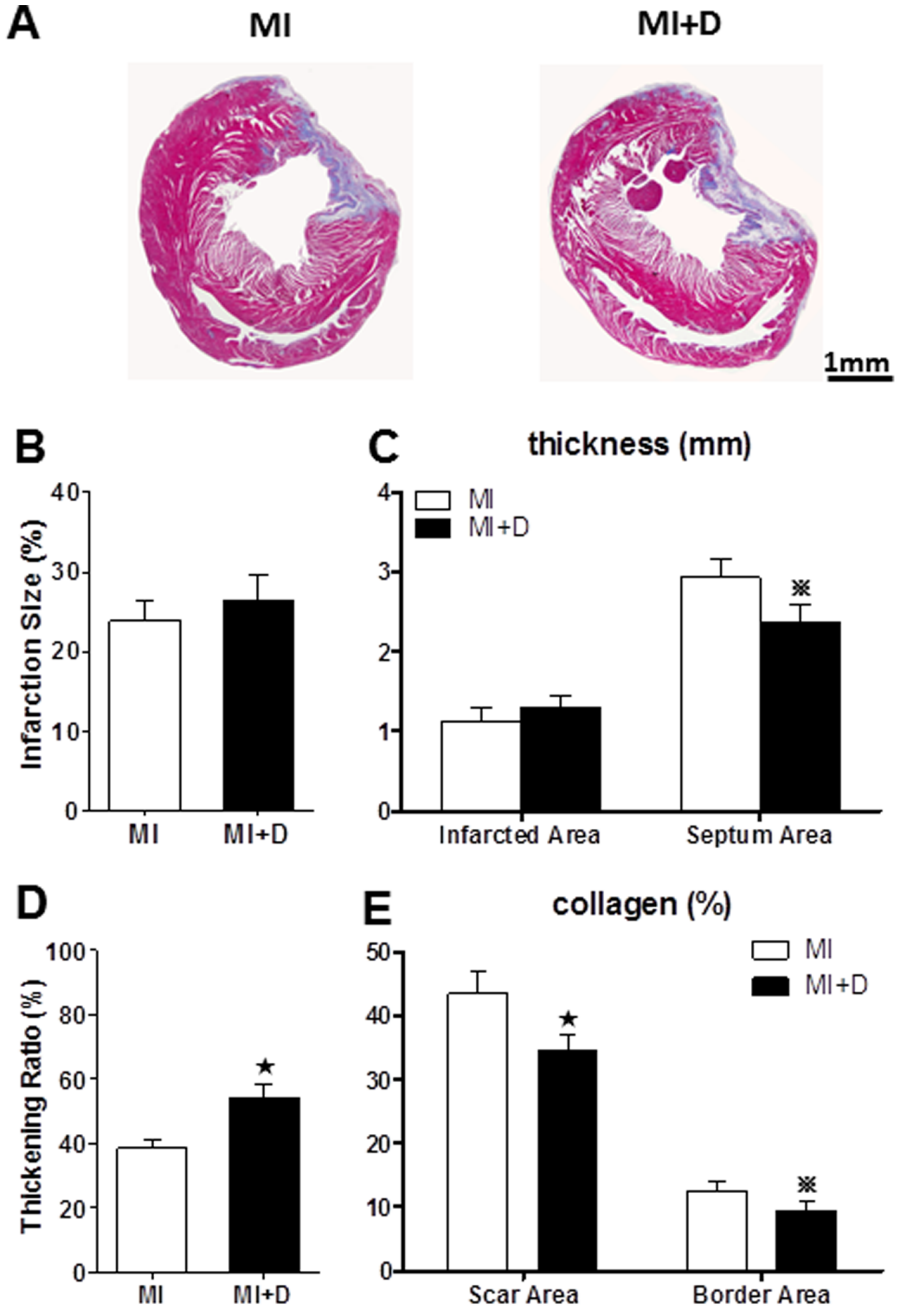
Infarct size and left ventricle morphometry. (A), Two representative Masson's trichrome-stained heart tissues from the MI group and MI+D group. Red and green indicate viable mass and scar tissue, respectively. (B), Quantitative analysis of infarction size. (C) Infarction wall thickness and septum wall thickness. (D), Thickening ratio. (E), Quantification of collagen from scar area and border area. n = 4 in each group, ^★^p<0.01 and ^
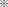
^p<0.05 vs MI group.

The infarction size was quantified as the percentage of scar tissue circumference on total LV circumference, which showed no significant changes in the MI+D group compared with the MI group (26.4±3.2% vs 23.9±2.5%, p = 0.14, Fig 8A and B). The infarction wall thickness also did not significantly differ between the groups (1.1±0.2 mm vs 1.3 ±0.2 mm, p = 0.23, Fig 8C). However, the MI group displayed a greater septum wall thickness than the MI+D group (2.9±0.2 mm vs 2.4±0.2 mm, p = 0.014, Fig 8 C). We also quantified the thickening ratio defined as the ratio of infarction wall thickness to noninfarcted wall thickness, and the MI+D group maintained a higher ratio than the MI+D group (54.3±4.2% vs 38.5±2.6%, p<0.01, Fig 8D). The collagen composition of the scar area and border area was relatively scant in the MI+D group compared with the MI group (Fig 8E).

## Discussion

The present study demonstrates that post-infarction rats treated with 4 weeks of CMS show higher indexes of depression-like behaviors. Compared with MI rats, the MI+D rats exhibit sympathetic hyperactivity and cardiac sympathetic hyperinnervation, decreased cardiac function, worse LV structure, more PVC, longer ventricular repolarization duration, shorter ERP, and increased susceptibility to VA. These findings indicate that chronic depression exerts hyperactivity of the sympathetic system and exacerbation of cardiac structural and electrical remodeling after MI.

### Depression and MI

Depression is associated with increased risk of cardiovascular events and mortality in patients with MI [4], [5], [18]. Although the exact mechanism remains unknown, it may be related to sympathetic hyperactivity and exacerbation of cardiac structural and electoral remodeling. To test this hypothesis, a rat model of depression was induced with CMS in this study. After receiving continuous various stresses for 4-week periods, the model rats exhibited decreased SP, reduced spontaneous locomotor activity and attenuated body weight gain compared with the controls. These findings are considered analogous to primary depressive-like symptoms, such as anhedonia, reduced exploration activity, psychomotor impairment and poor energy [19]. The lack of locomotor activity may reflect exercise intolerance caused by LV dysfunction. However, the amount of rearing, total distance travelled and time spent in peripheral zone were also decreased in the D group compared with the sham group, in which there was no significant difference in cardiac function; therefore, this phenomenon may largely reflect depressive status. Therefore, the CMS procedure is an effective method to produce the model of depression in this experiment, as previously reported [10], [13]. The survival rate was not significantly different among groups, which was inconsistent with clinical research, this may be associated with small sample size and short experimental period.

### Effect of depression on cardiac function

The close relationship between depression and subclinical cardiac dysfunction is accepted in the general population [20], and the rate of depression and the severity of depressive symptoms are significantly related to the severity of LV dysfunction in MI patients [21]. Consistent with clinical results, this study found that LV dimensions increased and FS decreased in the MI+D group compared with the MI group. Additionally, the MI+D group experienced a significant increase in the septum wall thickness and thickening ratio. The thickening ratio, which indexes both infarcted wall thickness and compensatory atrophy in remote myocardium, was higher in the MI+D group than the MI group. Interestingly, the proportion of collagen in the infarction area and the border area was lower in the MI+D group. Fiber repair is an important pathological process in an infarcted area, replacing active myocardium to maintain the integrity of cardiac structure and function; cardiac function will continue to deteriorate without enough replacement collagen fibers. The myocardial apoptosis induced by chronic mild stress may be a critical mechanism of adverse remodeling in MI+D hearts [12]. These findings indicated that the role of depression in accelerating the process of adverse cardiac structural remodeling could be explained by blunt compensation processes.

### Effect of depression on cardiac electrophysiology

Many clinical studies have suggested that depression is an independent risk of VA and sudden cardiac death [22], [23], and animal experiments have shown a link between depression and increased vulnerability to arrhythmias [24], [25]. Therefore, we hypothesized that depression would exacerbate the electrical instability and increase the onset of VA in a post-infarcted heart. Interestingly, the surface ECG showed that the occurrence of frequent PVC in the MI+D group but not in the MI or the other two groups. In addition, compared with the MI group, the MI+D group displayed elevated HR, longer QT duration, greater APD_90_ and shorter ERPs, which indicated that chronic depression may cause serious cardiac electrical remodeling in an infarcted heart. These changes may provide a substrate for conduction abnormalities and reentry—the most important mechanism of arrhythmias [26]. Importantly, and consistent with the above changes, the threshold value (S_1_-S_2_ interval) of VA inducibility was significantly higher in the MI+D group than the MI group, which suggests an increased susceptibility to VA after 4 weeks of CMS. There were also prominent alterations of cardiac electrophysiology in the D group; however, the VA could not be induced with S_1_-S_2_ stimulation method, which is inconsistent with previous findings [25] and could be explained by the difference in inducible method. In general, the current observations support the hypothesis that the association between depression and adverse prognosis involves an arrhythmia mechanism.

### The impact of depression on the sympathetic system

Previously studies suggested that the sympathetic system is persistently activated in patients with MI and plays a major role in its progression [27], [28], [29]. This type of autonomic nerve imbalance was also documented in depression disorders [30], [31], [32]. Therefore, the adverse effect of depression on an infarcted heart may be due to sympathetic activation. Consistent with this hypothesis, we found that the plasma level of E and NE were elevated in the MI+D rats compared with the MI rats, which indicated increased sympathetic activity. Additionally, cardiac sympathetic hyperinnervation in the MI border region was particularly prominent in the MI+D rats. Hyperactivity of the sympathetic system contributes to increased pulse rate and blood pressure, and damaged blood vessels. Blood then becomes viscous and prone to thrombosis. This increases the incidence of arrhythmias, the most common underlying mechanisms of myocardium injury and heart disease [33], [34], [35], [36].

### Limitations

Our study used a depression model that exhibits many aspects parallel to human depressive disorder. However, the physiological changes may differ in rats and humans. A total of 4 weeks of CMS was sufficient to induce depression-like behaviors in our study; however, post-infarction depression is usually a long-term process, and the period of experimental time needs to be extended further. Depression may have multi-pathogenic causes and may be related to disturbances of the hypothalamic pituitary axis, the renin angiotensin aldosterone system, the central nervous system, oxidative stress, increased inflammation, and the presence of genetic factors [2], [37]. Further studies are needed to clarify the other key pathophysiological mechanisms of interaction between depression and MI.

## Conclusions

In conclusion, these findings indicate that sympathetic hyperactivation may significantly contribute to the adverse effects of depression on cardiac function, morphology and electrical characteristics during MI, which highlights that depression disorders should be diligently screened and effectively prevented in MI patients.
